# Cost-effective Evaluation of an Educational Intervention based on Multiple Intelligences Versus Traditional Care to Improve Exclusive Breastfeeding in Mothers’ Clubs in Peru

**DOI:** 10.17533/udea.iee.v39n2e02

**Published:** 2021-06-15

**Authors:** Rubén Balabonce Chumpitaz Durand, Norma del Carmen Gálvez Díaz, Daniel Ángel Córdova Sotomayor

**Affiliations:** 1 Dentist Surgeon, Master’s. Research Professor. Email: rubencd@hotmail.com rubencd@hotmail.com; 2 Nurse, Master’s. Research Professor. Email: ncarmengd@crece.edu.pe ncarmengd@crece.edu.pe; 3 Social and Community Health, Master’s. Research Professor. Email: cordova.sotomayor.d@upch.pe cordova.sotomayor.d@upch.pe; 4 Universidad Señor de Sipán, Chiclayo, Peru. Universidad Señor de Sipán Universidad Señor de Sipán Chiclayo Peru; 5 Universidad Peruana Cayetano Heredia, Lima, Peru. Universidad Peruana Cayetano Heredia Universidad Peruana Cayetano Heredia Lima Peru

**Keywords:** breast feeding, health education, mothers, cost-benefit analysis, knowledge, lactancia materna, educación en salud, madres, análisis costo-beneficio, conocimiento, aleitamento materno, educação em saúde, mães, análise custo-benefício, conhecimiento

## Abstract

**Objective.:**

This work sought to evaluate the cost-effectiveness of two educational interventions to improve exclusive maternal breastfeeding in mothers’ clubs in Peru.

**Methods.:**

This is a retrospective, longitudinal descriptive study, which reviewed 113 information registries of mothers participating in a traditional educational intervention and 104 mothers with intervention based on multiple intelligences, evaluating the level of knowledge and exclusive maternal breastfeeding practices through observation visits.

**Results.:**

The intervention based on multiple intelligences achieved greater cost-effectiveness than the traditional intervention given that with lower investment, it managed to get 56.73% of the mothers to increase their knowledge and 68.27% to practice exclusive maternal breastfeeding from six months to more months in comparison with the traditional intervention in which 41.59% and 43.36% improved, respectively. Moreover, for its effective application, on every 100 mothers, its investment would result lower than the traditional program.

**Conclusion.:**

Upon applying satisfactorily the cost-effectiveness evaluation, as model to compare educational interventions on exclusive maternal breastfeeding, better results were determined with the intervention based on multiple intelligences compared with the traditional intervention, given that with lower investment, it was possible to benefit a greater number of mothers in their level of knowledge and practices of maternal breastfeeding.

## Resumen

**Objetivo.:**

Evaluar el costo-efectividad de dos intervenciones educativas para mejorar la lactancia materna exclusiva en clubes de madres en Perú.

**M**étodos.**:**

Es un estudio descriptivo, retrospectivo y longitudinal. Se hizo revisión de 113 registros de información de madres participantes de una intervención educativa tradicional y 104 madres con intervención basada en inteligencias múltiples, evaluando el nivel de conocimiento y las prácticas de lactancia materna exclusiva mediante visitas de observación.

**Resultados.:**

La intervención basada en inteligencias múltiples consiguió mayor costo-efectividad que la intervención tradicional debido a que, con menor inversión, se logró que el 56.73% de las madres incrementaran sus conocimientos y el 68.27% practicaran lactancia materna exclusiva de seis a más meses en comparación a la intervención tradicional en la cual el 41.59% y 43.36% mejoraran respectivamente. Además, para su aplicación efectiva, sobre cada cien madres, su inversión resultaría menor que el programa tradicional.

**Conclusión.:**

Al aplicar satisfactoriamente la evaluación costo-efectividad como modelo para la comparación de intervenciones educativas sobre lactancia materna exclusiva, se determinaron mejores resultados con la intervención basada en inteligencias múltiples en comparación con la intervención tradicional, pues, con una menor inversión, se logró beneficiar a una mayor cantidad de madres en su nivel de conocimientos y prácticas de lactancia materna.

## Resumo

**Objetivo.:**

Avaliar o custo-efetividade de duas intervenções educacionais para melhorar o aleitamento materno exclusivo em clubes de mães no Peru.

**Métodos.:**

Estudo descritivo, retrospectivo e longitudinal, baseado na revisão de 113 fichas de informações de mães participantes de uma intervenção educativa tradicional e 104 mães com uma intervenção baseada em inteligências múltiplas, avaliando o nível de conhecimentos e práticas da amamentação exclusiva por meio de visitas de observação.

**Resultados.:**

A intervenção baseada em múltiplas inteligências obteve maior custo-efetividade do que a intervenção tradicional, pois com menor investimento conseguiu que 56.73% das mães aumentassem seus conhecimentos e 68.27% praticasse o aleitamento materno exclusivo por 6 meses ou mais, em comparação com a intervenção tradicional em que 41.59% e 43.36% melhoraram respectivamente; além disso, para sua aplicação efetiva a cada cem mães, seu investimento seria inferior ao do programa tradicional.

**Conclusão.:**

Ao aplicar com sucesso a avaliação de custo-efetividade como modelo de comparação de intervenções educativas em aleitamento materno exclusivo, foram determinados melhores resultados a favor da intervenção baseada em múltiplas inteligências, em comparação à intervenção tradicional, constatando que, com menor investimento, A maior número de mães foi beneficiado em seu nível de conhecimento e práticas de amamentação.

## Introduction

Exclusive breastfeeding (EB) provides the natural, essential, and energy nutritional requirements needed by human beings during their first months of life.(1) Its consumption during the six months after birth guarantees optimal effectiveness to assimilate its multiple benefits that last during the following years of life.(2,3) Exclusive breastfeeding constitutes a priority in public health because, besides favoring the child’s growth and healthy development, it provides immunological protection, thus, reducing the risk of having infectious diseases and preventing conditions of malnutrition.(4) The benefits for the mother imply enhancing the affective and protective bond of their child, in addition to diminishing the risk of breast and ovarian cancer, post-partum hemorrhage, osteoporosis, and cardiovascular diseases.(5) Further, the impact on the economy is positive, becoming a savings by eliminating the use of complementary feeding and avoiding health costs due to possible causes of infant morbidity and mortality in all socioeconomic levels.(6)

In spite of seeking multiple benefits, some factors, like inadequate knowledge and practices, socio-economic aspects, difficulties in producing milk, rejection by the child, mother’s concerns, and health problems, could influence on early abandonment of EB.(7) The social factors associated with EB correspond to the young age of the pregnant women and to aspects regarding the composition, ontogenesis, integrality, and family functionality, while the cultural factors are related with customs and beliefs of the immediate environment. In that sense, family beliefs sometimes operate as cultural barriers.(3,8) In this respect, beliefs like “breastfeeding weakens the mother”, “EB is insufficient to satisfy the child’s hunger”, “it should be replaced by the infant formula bottle to better control the child’s feeding”, “breastfeeding damages the mother’s bodily image”, besides other erroneous ideas,(1,9) evidence and justify the need to implement educational interventions that promote maternal breastfeeding. Precisely, prior experiences have managed to prove their contribution. Researchers, like Hernández *et al*.,(10) were able to increase the level of knowledge and attitudes of a group of adolescents from Tenerife, demonstrating the effectiveness of an educational intervention on MB based on talks, videos, information leaflets, stories, and role playing. In another experience, Rojas *et al*.,(11) implemented educational workshops for pregnant women and mothers of children under two years of age in Venezuela, reporting a significant increase of their knowledge on themes of maternal breastfeeding. While Márquez *et al.,(*12) after applying the educational program “breastfeed me mommy” to a group of mothers of children under six months of age from Trujillo in Peru, reached significant effectiveness in the level of knowledge with respect to EB.

Although it is true that these experiences, as well as other similar experiences, have achieved favorable results, comparing intervention and control groups, the contribution of the current study lies in applying the evaluative approach of cost-effectiveness used in other disciplinary areas. According to its procedure, said evaluation is determined in function of the cost per unit benefitted, so that it permits projecting how much investment is needed to achieve greater effectiveness on a group of participants that should benefit from a preventive health intervention.(13) Thus, it is proposed as a valid experience to compare different EB promotion programs, which must consider baseline measurements, as well as control and follow up to identify strengths and weaknesses in timely manner and, thus, achieve higher effectiveness indices. This initiative seeks to benefit those responsible for designing, managing, developing, and evaluating prevention interventions in the field of nursing, socializing the scope of the research with competent authorities from the health sector and encouraging the possibility of consolidating public policies that promote a culture of preventive health in favor of EB. Regarding these considerations, the aim of the study corresponded to evaluating the cost-effectiveness of two educational interventions to improve exclusive breastfeeding in mothers’ clubs from Pimentel in Perú.

## Methods

A study was conducted with descriptive, retrospective and longitudinal design, based on the cost-effectiveness evaluation of two educational interventions on maternal breastfeeding, applied in 2018, with follow up in 2019, which required reviewing registries of knowledge and practices of MB obtained from two groups of pregnant women from the mothers’ clubs in the locality of Pimentel in Chiclayo, Lambayeque (Peru). The population was comprised of 217 registries of information from pregnant women who attended mothers’ clubs in four communal associations, distributed into 59 from the association “Los Jardines” and 54 from “Villa del Mar”, which participated in an educational intervention with the traditional methodology. In addition, the study had access to 55 registries of pregnant women from the association “Virgen de Fátima” and 49 from “Perla del Pacífico”, who participated in the educational intervention with the methodology based on multiple intelligences. Given that these were accessible amounts, the sample was represented by the entire study population.

In accordance with the selection criteria, the study included registries on knowledge and practices of MB from pregnant women from low socio-economic level who had no diseases that meant a risk for their health and that of the child, besides considering registries that were legible and with the required information for their control and follow up. Likewise, the study excluded registries whose evaluators did not undergo a prior calibration process, dispensing with registries of pregnant women who participated simultaneously in other training on maternal breastfeeding or who, at the moment of their evaluation and follow up, did not have an informed consent or that of their parents or legal guardians in case they were minors.

The information registries corresponded to a questionnaire on knowledge and a sheet for practical observation on MB. The questionnaire was used in previous studies, verifying its validation through expert judgment, a pilot test, and statistical reliability with Cronbach’s alpha coefficient (α = 0.86). This had 12 questions, each with five response options and a score of 1 was assigned for every correct item and 0 for each wrong answer, so that the level of knowledge was categorized as deficient (0 - 3 points), regular (4 - 6), good (7 - 9), and excellent (10 - 12). The practical observation sheet gathered information with respect to time of EB, verification of the technique used, and observation of any inconvenience that hindered the practice of EB. In addition, it was corroborated that the team of three nurses who conducted the surveys had passed satisfactorily through a prior calibration process (k = 0.81). A simple instrument was also used in which information was transcribed regarding the investment costs of both educational interventions, number of participants from both groups of pregnant women, and the dates of starting and ending the information registries.

Specifically, for the cost-effectiveness evaluation, it was necessary to divide the investment cost of each of the two educational interventions into the number of mothers who improved their knowledge and practices of EB in each group, so that it was possible to obtain the cost per unit benefited. This comparison permitted knowing which of the interventions required less investment to achieve a greater number of beneficiary mothers, which permits deciding between two health programs with different educational methodologies if greater preventive effectiveness were sought on a target population with the most productive use of resources. Gathering of information with respect to knowledge and practices of EB required three moments: a baseline registry and of immediate results conducted between April and July of 2018 and, then the follow-up registry between April and July of 2019, that is, one year after having applied the interventions. These were carried out in 2018 by three nurses previously trained by an expert instructor on traditional educational methodologies and on multiple intelligences, whose characteristics are described in Table 1.

**Table 1:** Comparison of the educational interventions with traditional and constructivist approaches to promote maternal breastfeeding

Comparison criteria	Traditional educational intervention	Intervention based on multiple intelligences
Facilitators from each educational intervention.	Team of three trained nurses from Universidad Señor de Sipán.	Team of three trained nurses from Universidad Señor de Sipán
Time of applicability of each educational intervention.	10 sessions (between April and July of 2018).	10 sessions (between April and July of 2018).
Hours per session	Between 2 and 3 hours.	Between 2 and 3 hours.
Place of implementation.	Mothers’ clubs from the communal associations “Los Jardines” and “Villa del Mar”.	Mothers’ clubs from the associations “Virgen de Fátima” and “Perla del Pacífico”.
Population benefited	113 pregnant women in 2018 (mothers in 2019).	104 pregnant women in 2018 (mothers in 2019).
Content of the interventions	EB: benefits. Nutritional contributions. Duration and frequency. Conservation and storage. Technique and correct position for breastfeeding. Importance of the mother-child bond and interaction. Signs of adequate suction. Problems associated with breastfeeding.	EB: Benefits. Nutritional contributions. Duration and frequency. Conservation and storage. Technique and correct position for breastfeeding. Importance of the mother-child bond and interaction. Signs of adequate suction. Problems associated with breastfeeding.
Educational materials	Leaflets, flip charts, videos, posters, brochures, mockups, whiteboard and markers of passive and static application.	Recycled materials that facilitated active participation with group, playful, and experiential dynamics.
Methodology	The active role was performed by the facilitators with educational and conversation-type demonstrative sessions in which the pregnant women listened, observed, and repeated the messages. Formulation of questions to verify that participants can repeat and memorize the messages from the educational sessions. Use of massive-type audio-visual educational material to propitiate individual responses.	The active role was performed by the pregnant women with dynamic educational and experiential sessions in which the mothers interacted actively. Formulation of questions to recover prior knowledge, verifying that they have found their own meaning of the messages. Use of dynamic, personalized and ludic material to propitiate group and individual dynamics.
Technique and collection instrument	Information registered through survey with validated questionnaire and sheet for practical observation of EB.	Information registered through survey with validated questionnaire and sheet for practical observation of EB.
Evaluation, control and follow up	Baseline evaluation (pretest), immediate evaluation and follow-up evaluation (post-test).	Baseline evaluation (pretest), immediate evaluation and follow-up evaluation (post-test).
Cost of investment	USD $765	USD $605

The traditional methodology is that which has been widely disseminated in the “Technical guide for advisory on maternal breastfeeding”, elaborated by the Health Promotion Direction of the Peruvian Ministry of Health.(14) The multiple intelligences methodology is based on the theory by Howard Gardner, who held that human intelligence is not limited to their intellectual capacity, but to a set of abilities and skills organized into eight types of intelligences. Table 2 shows the activities performed within the framework of this methodology.

**Table 2:** Activities conducted during the educational intervention based on multiple intelligences

Multiple intelligences	Activities conducted
Linguistic-verbal intelligence	Stories, riddles, acrostics and popular sayings were elaborated related with the EB practice, healthy eating, and importance of pregnancy control. Oral interventions, debates, exchange of opinions, expositions and questions to verify their progress were also encouraged.
Musical intelligence	Songs were created by changing the lyrics of known songs, adapting them to EB promotion. Healthy eating and periodic visits to pregnancy control were promoted. Besides organizing and carrying out educational activities with respect to an artistic musical show with song and dance alluding to EB promotion.
Logical-mathematical intelligence	The mothers were asked to participate in practical exercises that propitiated the calculation of time and frequency of EB, besides making a list of healthy foods organized according to the nutritional content, size of ration, cost, and access to establishment where these are sold. Se les asked, additionally, to follow the practical and logical order of EB and to organize probable dates for their follow up and control.
Bodily-kinesthetic intelligence	Simulations were made with the technique and logical sequence of EB, controlled by the nursing staff and supervised by the expert instructor. Participants recognized the safe settings and services where they can carry out EB and engage in controlled physical activity. Activities were conducted, like simple Taiichi competitions and games alluding to EB.
Intrapersonal intelligence	Reflection on the importance of evaluating the child’s health and their own and analyzing how they would feel after their EB practices, propitiating the affective bond with their child. They commented how they felt every time they attend medical controls and assumed the commitment to continue and respect health care.
Interpersonal intelligence	Helping each other to improve their simulated technique of EB. Groups were created to solve situations posed by the instructor, related with health care during pregnancy. Through games and dynamics, they appraised teamwork and recognized the importance of the family’s participation to achieve healthy behaviors.
Visual-spatial intelligence	Comparison of healthy foods with foods harmful for their condition as pregnant women. The study verified and recorded the types of foods sold in the establishments and near to their immediate environment. Identification of settings to engage in physical activities as part of their healthy lifestyles.
Naturalistic intelligence	Dynamics were carried out to promote and propitiate safe and healthy environments to practice EB. Analysis was made of the importance of consuming natural foods (water, proteins, fruits and vegetables) rather than artificial and processed foods.

With regards to bioethical principles, the research complies with the Helsinki International Declaration, besides adhering to the guidelines of the Code of Ethics for Research by the Vice-rectory of Research at Universidad Señor de Sipán, approved through Directory Resolution N°199 - 2019 / PD - USS, having been a requirement that the women participating in the study had granted their corresponding signed informed consent. The Kolmogorov-Smirnov test was applied in the data processing to demonstrate compliance of the assumption of normality in the data distribution. The cost-effectiveness evaluation used simple descriptive statistics calculations, like sums, averages, and percentages, based on the ratio of investment costs and the effectiveness reached according with the number of mothers benefitted with better MB knowledge and practices. With the purpose in mind, the Wilcoxon test was used for the before and after comparison with respect to the level of knowledge in each group. Thereafter, the analysis included the comparison of both groups through the Mann-Whitney U test, using 95% confidence interval. The data processing used the SPSS program version 22.

## Results

According with the information registries obtained from the mothers’ clubs from the Communal Associations in Pimentel, 11 were excluded because they were minors whose parents refused the informed consent, selecting 217 information registries of pregnant women. Table 3 shows the distribution of the women in the study according with their stage of life at the moment of the pregnancy, identifying that 48.67% of the pregnant women were adolescents and 51.33% of the pregnant women were adults who participated in the group of educational intervention with traditional methodology in comparison with 47.12% of the pregnant women in adolescent stage and 52.88% of the adult pregnant women from the group of intervention based on multiple intelligences. Moreover, according to their experience as mothers, it was evidenced that, in both educational intervention groups, there were primiparous mothers in higher percentage than women who mothers for the second or more times.

**Table 3:** Distribution of mothers participating in both educational interventions according to the life stage as pregnant women and experience as mothers

Life stage of the pregnant woman	Experience as mothers	Experience as mothers	Experience as mothers	Experience as mothers	Experience as mothers	Experience as mothers	Experience as mothers	Experience as mothers	Experience as mothers	Experience as mothers	Experience as mothers	Experience as mothers
Life stage of the pregnant woman	Traditional intervention	Traditional intervention	Traditional intervention	Traditional intervention	Traditional intervention	Traditional intervention	Intervention with Multiple intelligences	Intervention with Multiple intelligences	Intervention with Multiple intelligences	Intervention with Multiple intelligences	Intervention with Multiple intelligences	Intervention with Multiple intelligences
Life stage of the pregnant woman	**(*n* = 113)**	**(*n* = 113)**	**(*n* = 113)**	**(*n* = 113)**	**(*n* = 113)**	**(*n* = 113)**	**(*n* = 104)**	**(*n* = 104)**	**(*n* = 104)**	**(*n* = 104)**	**(*n* = 104)**	**(*n* = 104)**
Life stage of the pregnant woman	Primiparous	Primiparous	For a second time	For a second time	3 to more times	3 to more times	Primiparous	Primiparous	For a second time	For a second time	3 to more times	3 to more times
Life stage of the pregnant woman	*n*	%	*n*	%	*n*	%	*n*	%	*n*	%	*n*	%
Early adolescence	7	6.19	0	0	0	0	8	7.69	0	0	0	0
Medium adolescence	11	9.73	2	1.77	0	0	9	8.65	0	0	0	0
Late adolescence	28	24.78	7	6.19	0	0	22	21.15	9	8.65	1	0.96
												
Young adult	22	19.47	17	15.04	4	3.54	19	18.27	21	20.19	2	1.92
Medium adult	5	4.42	4	3.54	3	2.65	4	3.85	5	4.81	2	1.92
Mature adult	0	0	1	0.88	2	1.77	0	0.00	1	0.96	1	0.96

Regarding mothers who benefitted in increasing their level of knowledge with respect to MB, Table 4 shows that mothers with a good level went from 4.8% to 30.8% after the intervention with multiple intelligence in comparison with the traditional group that went from 6.2% to 23%, while those who achieved excellence level were 11.5% from the first group and 5.3% from the second. After the paired analysis, it was concluded that mothers who benefitted, going from good to excellent level, from regular to good, and from deficient to regular, corresponded to 47 from the traditional group and 59 from the group with MI; finding significant difference according to the Mann Whitney U test (*p* = 0.002). Additionally, upon determining the difference of 41.5% (47/113) versus 56.7% (59/104), it was possible to obtain (*p* = 0.0019), confirming statistical significance.

**Table 4:** Knowledge of maternal breastfeeding by mothers before and after their participation in both educational interventions

Level of knowledge	Experience as mothers	Experience as mothers	Experience as mothers	Experience as mothers	Experience as mothers	Experience as mothers	Experience as mothers	Experience as mothers
Level of knowledge	Traditional intervention	Traditional intervention	Traditional intervention	Traditional intervention	Intervention with multiple intelligences	Intervention with multiple intelligences	Intervention with multiple intelligences	Intervention with multiple intelligences
Level of knowledge	**(*n* = 113)**	**(*n* = 113)**	**(*n* = 113)**	**(*n* = 113)**	**(*n* = 104)**	**(*n* = 104)**	**(*n* = 104)**	**(*n* = 104)**
Level of knowledge	Pretest	Pretest	Post-test	Post-test	Pretest	Pretest	Post-test	Post-test
Level of knowledge	*n*	%	*n*	%	*n*	%	*n*	%
Deficient	59	52.2	12	10.6	62	59.6	3	2.9
Regular	47	41.6	69	61.1	37	35.6	57	54.8
Good	7	6.2	26	23.0	5	4.8	32	30.8
Excellent	0	0	6	5.3	0	0	12	11.5
Total	113	100	113	100	104	100	104	100
Median	3	3	4	4	3	3	5	5
Mean	3.67	3.67	5.27	5.27	3.5	3.5	6.07	6.07
Wilcoxon	*p <*0.0001	*p <*0.0001	*p <*0.0001	*p <*0.0001	*p* <0.0001	*p* <0.0001	*p* <0.0001	*p* <0.0001
Benefitted	47	47	47	47	59	59	59	59
Mann Whitney U	*p* = 0.384 (pretest)	*p* = 0.384 (pretest)	*p* = 0.384 (pretest)	*p* = 0.384 (pretest)	*p* = 0.002 (post-test)	*p* = 0.002 (post-test)	*p* = 0.002 (post-test)	*p* = 0.002 (post-test)
Mann Whitney U	No difference between intervention groups	No difference between intervention groups	No difference between intervention groups	No difference between intervention groups	Significant difference between intervention groups	Significant difference between intervention groups	Significant difference between intervention groups	Significant difference between intervention groups

Kolmogorov Smirnov =<0.0001 (no normal distribution in any case)

Regarding the mothers who benefitted with the best MB practices, Table 5 evidences that 68.27% of the mothers who participated in the intervention with multiple intelligences practiced EB from six to more months in comparison with 43.36% of the group with traditional intervention, finding a higher percentage of mothers with MB interrupted prior to six months in the latter group. Additionally, from this table it may be determined that 63.46% of the mothers in the group with multiple intelligences reached an optimal MB technique against 38.05% in the traditional group. Difficulties that propitiated interrupting EB, in both groups, were unfavorable family environment and, more frequently, job conditions and mothers’ poor nutrition.

**Table 5:** Maternal breastfeeding practices after the mothers participated in the educational interventions

MB practices	Groups of pregnant women	Groups of pregnant women	Groups of pregnant women	Groups of pregnant women
MB practices	Traditional intervention	Traditional intervention	Intervention with MI	Intervention with MI
MB practices	*n*	%	*n*	%
Duration of MB				
Interrupted between 1 and 3 months	7	6.19	4	3.85
Interrupted between 3 and 6 months	57	50.44	29	27.88
Up to 6 months	49	43.36	71	68.27
MB technique				
Insufficient	11	9.73	7	6.73
Regular	59	52.21	31	29.81
Optimal	43	38.05	66	63.46
Difficulties for MB				
Mother's poor nutrition	14	12.39	2	1.92
Unfavorable family environment	23	20.35	11	10.58
Job condition	11	9.73	13	12.50
None	65	57.52	78	75.00

With respect to the cost-effectiveness evaluation, Table 6 shows that with an investment of USD $605 for a group of women with the intervention based on multiple intelligences, although lower than the USD $765 for the cost of the traditional intervention, a greater number of mothers was benefitted: 59 women increased their level of knowledge and 71 demonstrated EB practices of six to more months compared with the 47 mothers who increased their knowledge and 49 who improved their practices with the traditional intervention.

With respect to the level of knowledge, it is evidenced that with intervention using MI, when dividing its investment cost between the number of mothers benefitting, cost per unit of USD $10.25 was obtained, so that if effectiveness were sought in 100 mothers, the investment would have to be USD $1025, that is, a lower investment than with the traditional intervention, which would cost USD $1628 to benefit the same number of mothers: thus, needing an additional USD $603 to obtain effectiveness achieved by the other program. The results of cost-effectiveness with respect to EB practices also indicated an advantage in favor of the beneficiaries of care based on multiple intelligences upon having determined that to intervene 100 mothers, the investment cost of USD $852 would be lower than the USD $1561 needed by the traditional program, requiring an additional USD $709.

**Table 6:** Cost-effectiveness valued in US Dollars in both educational interventions to promote MB knowledge and practices

Educational interventions	Total cost of interventions	Proportion of mothers benefitted	Cost- effectiveness per unit benefitted	Cost-effectiveness for 100 mothers
Traditional intervention	$ 765			
Knowledge		47 (41.59% of 113)	$ 16.28	$ 1628
MB practices		49 (43.36% of 113)	$ 15.61	$ 1561
MI intervention	$ 605			
Knowledge		59 (56.73% of 104)	$ 10.25	$ 1025
MB practices		71 (68.27% of 104)	$ 8.52	$ 852

It must be indicated that the favorable cost-effectiveness results for the intervention with multiple intelligences are not due to greater investment, but to a more effective ratio of the investment cost with respect to the higher number of mothers benefitted.

## Discussion

In accordance with that estimated by the Pan-American Health Organization (PAHO) and the World Health Organization (WHO),(2,15) EB practices could save each year approximately 220 000 lives around the world due to the antibodies it contains and which protect the infants from frequent diseases during childhood. Madrid *et al*.,(5) indicate that its consumption contributes to diminishing the risk of having diarrheic and respiratory diseases, as well as asthma, type-I diabetes, sudden death, high blood pressure, and leukemia. Studies by Oyarzún *et al*.,(16) found association between antecedent of inconclusive MB and obesity and metabolic syndrome in school-age children. In another research, Binda *et al*.,(17) report that interrupting EB delays the child’s psychomotor and cognitive development. This reality becomes worrisome when the POHA(15) reveals that in the Region of the Americas only 38% of infants receive EB for up to six months.

The Direction of Health Promotion of the Peruvian Ministry of Health(18) reports that the EB practice is far below the values considered optimal by the WHO; for this reason, in 2017 the commission of experts from the Ministry of Health(14) elaborated the “Technical guide for advisory on maternal breastfeeding” for the purpose of establishing the conceptual, methodological, and instrumental criteria to conduct the advisory on MB that propitiates the exercise of the right of mothers to breastfeed their children for up to two years of age or more. The recommendations in this technical guide, as well as those established in studies by Pérez and Valdés(19) and Ortega,(20) are based on MB being considered a behavior susceptible to being learned, so that it is necessary to provide to the mothers training settings, given that by still being in a vulnerable stage, they require physical and emotional guidance with optimal support from the health system and the family environment. Within this context, experiences and investigations are widely justified with respect to EB promotion.

Regarding the results obtained in this work, participation is evidenced of nearly 50% of pregnant women in adolescent stage, whose condition, according to Pinilla *et al*.,(21) exposes them to their inexperience to assume, within a short period, the self-care of their health and that of their child, as well as the affective relations with the child and the family environment; challenges that lead them to endure a strong emotional burden, thus, becoming a risk factor for healthy MB. With respect to the effectiveness reached, better results were determined in favor of the intervention based on multiple intelligences, managing to benefit 56.73% of the mothers in their level of knowledge, compared to 41.59% of the participants benefitted from the group with traditional intervention. Similar results were reported by Rojas *et al*.,(11) who achieved 28.8% of the participants with deficient level knowledge changing to a good level in 60.8% of 1,132 pregnant women and mothers from different regional states of Venezuela after attending educational and participative workshops on MB. Another study by Rodríguez *et al.,*(22) managed to get the level of good knowledge to go from 21.81% to 70.9%, after an intervention based on six educational sessions of MB, applied to a group of 55 mothers from a poly-clinical institution in Camagüey, Cuba. In a similar experience, Melo *et al*.,(23) upon implementing an educational program with 201 pregnant women from a Maternal Hospital in Fortaleza, Brazil, reported an increase from 74.1 to 79.1 points (of 100 possible) in the level of knowledge, after an educational intervention based on a flip-chart about MB in comparison with the control group that dropped from a level of 72.8 to 70.7 points.

In more encouraging results, Márquez *et al.,*(12) after applying the educational program “breastfeed me mommy” to a group of 55 mothers of children under six months of age from a health center in Trujillo, Peru, managed to change the level of knowledge with respect to MB, from 93.4% with intermediate level to 100% with high level. Guerra *et al*.,(24) also reported a significant impact when intervening with an educational strategy on MB based on participative dynamics applied in 1,343 future mothers from a poly-clinical institution in Cuba, so that from an initial result with 47.3% of women with low level of knowledge managed to get 96.7% to reach a high level after the intervention; while Hernández *et al.,*(10) upon implementing an educational intervention on MB based on talks, videos, informative leaflets, participative stories, and role playing in 970 adolescents from Tenerife, managed to increase the level of knowledge from 48.75% (3.9 points of 8 possible) to 86.25% (6.9 points) after the experience, compared to a control group that only went from 3.8 to 4.3 points.

With respect to EB practices, this study determined that 43.36% of the mothers with traditional intervention prolonged EB from 6 months to more, in comparison with the intervention based on MI, where better results were found upon evidencing that 68.27% of them practiced breastfeeding during that period of time. Similar results were revealed in the research by Melo *et al*.,(23) reporting that EB practices up to 6 months were conducted by 64.31% of the participants from the educational intervention group, against 56.96% by a control group. Rodríguez *et al*.,(22) unlike prior results, found greater effectiveness when reporting that mothers with EB practices up to 6 months went from 32.72% before the intervention to 76.36% after it. Reaching greater impact, Guerra *et al.,(*24) reported that 90.42% of the mothers evidenced EB practices up to 6 months after an educational intervention. In turn, Escalona *et al*.,(25) also found good results when implementing an intervention based on Leininger’s Transcultural Nursing theory on 30 mothers cared for in a Hospital in Carabobo, Venezuela, managing to improve the EB practices from 44.9% to 85.4% of the participants after the intervention.

It must be indicated that among the difficulties limiting the extension of EB for an optimal time and which coincided with López *et al*.,(7) and Quispe *et al.,*(9) were the mother’s poor nutrition, unfavorable family environment, job conditions, and young age. Regarding the adequate MB technique, following the recommendations by the Peruvian Ministry of Health (14, 18) and coinciding with studies by Melo *et al*.,(23) 63.46% of the mothers from the group with MI and 38.05% from the traditional group evidenced favorable signs of bonding between mother and child and a comfortable and sustained position that propitiated optimal suction for the child, with these groups of mothers practicing EB up to 6 months.

As observed, reference studies coincide in having determined the effectiveness of the educational interventions from the comparison of an intervention group with a control group, unlike the present research in which both were evaluated with different methodological bases, highlighting as additional contribution that of having tested the applicability of the cost-effectiveness approach as a valid alternative to compare health promotion programs.

The findings, herein, evidence the contribution to improve the nursing practice, given that they propitiate the likelihood of being involved in health promotion programs, appealing to educational methodologies that enrich and guarantee the effectiveness of health education processes, whose interventions can be evaluated from a cost-effectiveness analysis as a valid model that can contribute to measuring the real impact of programs promoting maternal breastfeeding, as well as other thematic axes related to preventive nursing care. Moreover, a limitation that hindered follow up of the mothers was the difficulty to locate them in their homes because, in many cases, they had begun their work activity, which meant greater time dedicated to culminate the follow up and evidenced one of the reasons why they had suspended EB. Another difficulty is the lack of experiences directly related with the present study, which could have enriched the discussion further. Due to this, additional scientific work must continue to address that line of research, making it necessary to socialize the findings with pertinent authorities to propitiate healthy EB promotion policies that manage to establish a culture of preventive health in our country, especially in vulnerable communities.

Finally, careful control of possible bias was guaranteed after conducting a strict selection process of the criteria of the information registries, so that the results to determine the costs and effectiveness were attributed exclusively to the educational interventions and not to other factors that could have influenced.

This study concludes that when applying satisfactorily the cost-effectiveness evaluation as model to compare educational interventions on EB, better results were achieved in favor of the intervention based on multiple intelligences, finding that, with a lower investment, it was possible to benefit a greater number of mothers in their level of knowledge and maternal breastfeeding practices compared with a traditional educational intervention.
